# Isavuconazole therapeutic drug monitoring in real-world clinical practice: an age-stratified analysis of determinants

**DOI:** 10.3389/fphar.2026.1779322

**Published:** 2026-04-30

**Authors:** Zhaoyi Tan, Linlin Lv, Wenjing Gu, Na Zhang, Dengfeng Huang, Beibei Liang, Bei Yan, Fan Jiang, Yun Cai

**Affiliations:** 1 The Phase I Clinical Trial Laboratory, The First Medical Center of the PLA General Hospital, Beijing, China; 2 Center of Medicine Clinical Research, Department of Pharmacy, Medical Supplies Center, PLA General Hospital, Beijing, China; 3 Department of Hematology, Beijing Jingdu Children’s Hospital, Beijing, China; 4 Department of Hematology, China Aerospace Science & Industry Corporation 731 Hospital, Beijing, China

**Keywords:** elderly patients, invasive fungal infection, isavuconazole, pediatric patients, therapeutic drug monitoring

## Abstract

**Objectives:**

Although therapeutic drug monitoring (TDM) for isavuconazole (ISA) is not routinely recommended, its utility in specific populations remains debated. This study aimed to elucidate the need for ISA TDM and to identify the specific patient groups who might benefit from it.

**Methods:**

We retrospectively analyzed 48 patients (13 pediatric, 19 non-elderly, 16 elderly) who underwent ISA TDM. Clinical characteristics, TDM results, dose adjustments, and outcomes were collected.

**Results:**

A total of 179 trough measurements were obtained. Pediatric patients had a lowest median C_trough_ of 2.6 mg/L and the highest interindividual variability (51.6%), particularly lower-weight pediatric patients were at an increased risk of underexposure. The median ISA C_trough_ was significantly lower in elderly compared to non-elderly patients (2.9 vs. 4.3 mg/L, *P* < 0.001). TDM led to 21 dosage adjustments and substantial proportion of patients had C_trough_ outside the therapeutic range (2–5 mg/L): 46.7% of pediatric patients, 48.9% of non-elderly patients, and 36.5% of elderly patients. Female sex, Intensive Care Unit (ICU) admission, and renal replacement therapy (RRT) were associated with lower levels in elderly patients.

**Conclusion:**

TDM helps optimize dosing, especially in populations with high variability such as pediatric patients. Female sex, ICU admission, and RRT are important factors associated with lower exposure in elderly patients.

## Graphical Abstract

**Figure:**
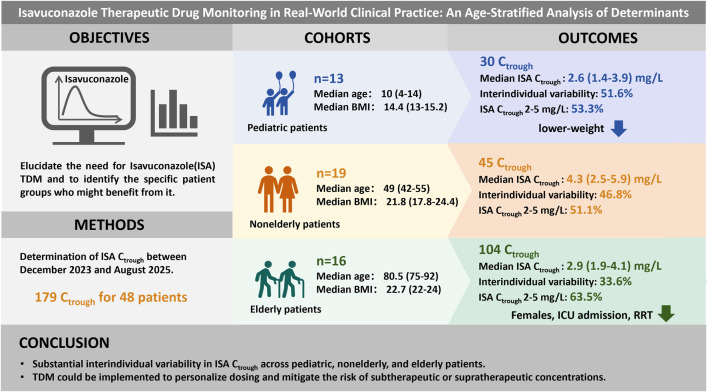

## 1 Introduction

Isavuconazole (ISA) is a triazole antifungal agent approved for the treatment of invasive aspergillosis and mucormycosis (Thompson et al., 2024). ISA is characterized by high oral bioavailability (98%), high plasma protein binding, a long elimination half-life (100–130 h), and hepatic metabolism by CYP3A4 and CYP3A5 (Lewis et al., 2022; Falci and Pasqualotto, 2013). Evidence from the SECURE and VITAL trials demonstrates the efficacy and safety of ISA for the treatment of adults with invasive fungal infections caused by *Aspergillus* spp. or other filamentous fungi, including mucormycosis (Maertens et al., 2016; Marty et al., 2016). A recent meta-analysis with trial sequential analysis further indicated that ISA had a significantly lower incidence of drug-related AEs (RR: 0.70, 95% CI: 0.61–0.81) and discontinuation due to drug-related AEs (RR: 0.56, 95% CI: 0.39–0.82) compared with voriconazole (Weng et al., 2025). ISA can also be safely and effectively administered to patients even after the failure of other azole therapies, such as voriconazole and posaconazole (Nwankwo et al., 2022; DiPippo et al., 2019; Cattaneo et al., 2019). ISA has been available in China since 2022. Studies indicate that, despite its higher unit price, it offers superior safety and a lower total treatment cost compared to voriconazole, owing to a lower maintenance dose and reduced monitoring and adverse event management cost (Han et al., 2023; Liu et al., 2025). It therefore represents a more economical alternative worthy of broader clinical application.

Although therapeutic drug monitoring (TDM) is standard practice for optimizing treatments with triazole antifungals like voriconazole, posaconazole, and itraconazole (McCreary et al., 2023; Hamada and Yagi, 2025; Ashbee et al., 2014; John et al., 2019), its role in managing ISA therapy remains debated. Initially, ISA was thought not to require TDM owing to its predictable pharmacokinetic profile, which includes high oral bioavailability, linear pharmacokinetics, modest drug-drug interaction potential, and a favorable hepatic safety profile (Lewis et al., 2022; Bolcato et al., 2022). However, emerging evidence supports its potential utility in specific populations, especially pediatric patients (Zimmermann et al., 2022; Borman et al., 2020; Luque et al., 2024), the elderly patients (Stemler et al., 2023; Perez et al., 2023), and critically ill patients (Herranz-Bayo et al., 2024; Perez et al., 2023; Höhl et al., 2022; Kosmidis et al., 2020). This is because different physiological and pathological conditions make the pharmacokinetics of ISA difficult to predict. There is no international consensus about the ideal target range for ISA trough concentrations (C_trough_), in general, and particularly not special populations such as pediatric patients and elderly patients.

Thus, we conducted a retrospective study using real-world TDM data from three tertiary care centers in China. We aimed to investigate ISA exposure stratified by age, evaluate the impact of patient characteristics on C_trough_, and assess the clinical value of TDM in guiding individualized dosing. Our findings may provide useful insights for optimizing ISA therapy in Chinese populations.

## 2 Materials and methods

### 2.1 Study design

TDM for ISA is available in very few centers across the country. This was a multicenter retrospective cohort study that included C_trough_ samples assayed at Chinese PLA General Hospital between December 2023 and August 2025. These samples were sourced from Beijing Jingdu Children’s Hospital, China Aerospace Science and Industry Corporation 731 Hospital, and the Chinese PLA General Hospital.

### 2.2 Data collection

TDM data collected comprised the sampling date and plasma drug concentration. Patient information concerned demographic (age, sex, weight, and body mass index (BMI)), clinical (department, underlying disease, microbiological examinations and use of renal replacement therapy (RRT)/extracorporeal membrane oxygenation (ECMO)), biological parameters (aspartate aminotransferase (AST), alanine aminotransferase (ALT), gamma glutamyltransferase (GGT), alkaline phosphatase (ALP), total bilirubin (TBil), total protein, creatinine (Cr), albumin (ALB), and lactate dehydrogenase (LDH) levels), and pharmaceutical (dosage and administration, date of initiation of treatment, and treatment response). The response to ISA was defined as a complete or partial response, stable disease, or disease progression (Denning et al., 2002). Information on adverse effects (ranked according to Common Terminology Criteria for Adverse Events, version 5.0) was collected for all patients.

### 2.3 TDM of ISA

ISA blood concentrations were determined 30 min before the next ISA dose was administered (labeled as ISA C_trough_). TDM was performed if deemed appropriate by the treating physician. The reasons for conducting TDM include suspected lack of efficacy, unexplained toxicity, ICU admission, as well as pediatric and elderly patients. Uncertainties remain regarding the optimal therapeutic range for ISA. Recent studies have identified various thresholds for potential toxicity, including C_trough_ of 4.6 (Kosmidis et al., 2020), 4.87 (Furfaro et al., 2019), 4.8 (Gomez-Lopez et al., 2023), 5 (Risum et al., 2021), 5.13 (Furfaro et al., 2019), and 5.86 mg/L (Huang et al., 2025). The 2022 guideline of the Infectious Diseases Working Party (AGIHO) of the German Society for Haematology and Medical Oncology (DGHO) recommends a target therapeutic range of 2–5 mg/L for TDM of ISA (Stemler et al., 2023). Accordingly, 2–5 mg/L is proposed as an appropriate reference range for safe and effective treatment (Tan et al., 2025), while the final decision on dose adjustment should be based on the clinician’s comprehensive judgment.

### 2.4 Measurement of ISA levels

Plasma ISA concentrations were determined using a liquid chromatography-tandem mass spectrometry (LC-MS/MS) method. Briefly, ISA quantification in plasma ranged from 0.2 to 16 mg/L. The inter-batch accuracy and precision ranged from 90.00% to 108.31% and 2.20%–8.04%, respectively. The intra-batch accuracy and precision ranged from 92.28% to 100.74% and 0.31%–3.00%, respectively.

### 2.5 Statistical methods

We stratified the population into 3 age groups: <18 years (pediatric patients), 18–65 years (non-elderly patients), and >65 years (elderly patients). Inter-individual variability was assessed by calculating the coefficient of variation (CV) of the mean ISA C_trough_ for all patients. A total of 4 clinical covariates were included into the univariable analysis to identify possible correlations with ISA C_trough_. For categorical variables (including sex, RRT, and ICU), comparisons between groups were performed using Mann-Whitney test. For continuous variables BMI, comparisons between groups were performed using Pearson test. Two sets of analyses were performed, the first focused on ISA C_trough_ and the second on the C_trough_ of ISA normalised by the daily dose (for pediatric patients: C_trough_/Dose*weight*5.4, for adult patients: C_trough_/Dose*200). The dose normalization was based on the fact that most pediatric patients received a dosage regimen of 5.4 mg/kg/day (up to a maximum of 200 mg), whereas adult patients (including elderly patients) received a regimen of 200 mg/24 h. A linear mixed-effects model was used to assess the associations between biochemical variables (ALT, AST, GGT, TBil, Cr, ALB) and ISA C_trough_. Statistical analysis was performed using SPSS version 27 (IBM Corp., Armonk, NY, United States of America). A *P*-value < 0.05 was considered statistically significant.

## 3 Results

### 3.1 Study population

48 patients had at least one determination of ISA blood levels and were included in the study, comprising 13 pediatric patients, 19 non-elderly patients and 16 elderly patients, males (72.9%) accounted for the majority. The baseline characteristics of the patients are summarized in Table 1. In the pediatric patients, the median age was 10 years (IQR 4–14) and the median BMI was 14.4 kg/m^2^ (IQR 13–15.2). The median age of non-elderly patients and elderly patients was 49 years (IQR 42–55) and 80.5 years (IQR 75–92) respectively, and their median BMI were 21.8 kg/m^2^ (IQR 17.8–24.4) and 22.7 kg/m^2^ (IQR 22–24) respectively. The most common underlying conditions were hematological malignancy (52.1%), cardiovascular disorders (33.3%) and liver disease (33.3%). In adults, 27 patients (77.1%) had a proven invasive fungal infection of which 16 (59.3%) had proven aspergillosis, 9 (33.3%) had proven mucormycosis and 2 (7.4%) had proven aspergillosis and mucormycosis co-infection. More patients are treated with oral administration (64.6%). The median duration of ISA therapy was 27.5 days (IQR 14–49.3) for non-elderly patients and 40.5 days (IQR 19.8–59) for elderly patients. 6 critically ill patients required RRT, none of the patients underwent ECMO support.

**TABLE 1:** Baseline and treatment characteristics of the patients.

Parameter	Pediatric patients (n = 13)	Adults		
		Total (n = 35)	Non-elderly patients (n = 19)	Elderly patients (n = 16)
Characteristic^a^				
Age, years	10 (4–14)	60 (48–79)	49 (42–55)	80.5 (75–92)
Male, n (%)	9 (69.2)	26 (74.3)	16 (84.2)	10 (62.5)
Weight, kg	32.8 (11.5–40.3)	64 (51.5–70.5)	63 (49–74)	64.5 (59.4–67.8)
BMI, kg/m^2^	14.4 (13–15.2)	22.5 (20–24.2)	21.8 (17.8–24.4)	22.7 (22–24)
Hospitalisation units, n (%)				
Haematology	13 (100)	7 (14.6)	6 (31.6)	1 (6.2)
ICU^e^	0 (0)	17 (48.6)	10 (52.6)	7 (43.8)
Underlying disease, n (%)				
Hematological malignancy	13 (100)	12 (34.3)	8 (42.1)	4 (25)
Cardiovascular disorders^b^	0 (0)	16 (45.7)	5 (26.3)	11 (68.8)
Liver diseases	4 (30.8)	12 (34.3)	9 (47.4)	3 (18.8)
Diabetes mellitus	0 (0)	13 (37.1)	7 (36.8)	6 (37.5)
Hypertension	0 (0)	13 (37.1)	2 (10.5)	11 (68.8)
Others^c^	0 (0)	7 (20)	3 (15.8)	4 (25)
RRT^d^, n (%)	0 (0)	6 (17.1)	3 (15.8)	3 (18.8)
Biological parameters at first ISA TDM				
AST (U/L)	45 (20–85.8)	25.7 (19.2–45.9)	35.9 (19.2–67)	24.7 (19.3–30.4)
ALT (U/L)	59 (29.5–121.9)	23 (13.3–44.8)	31.6 (16.3–73)	17.5 (11.1–26.2)
GGT (U/L)	214.1 (58–317.5)	137.4 (47.3–370.4)	137.4 (94.1–445.9)	132.5 (32–294.2)
TBil (µmol/L)	14.1 (6.8–46.3)	8.8 (5.9–12.9)	8.7 (6.5–9.3)	9.9 (6.9–17.9)
Cr (µmol/L)	32.4 (21.7–38.4)	76 (59.6–92.5)	66.4 (58.1–91.5)	80.5 (65.8–95)
LDH (U/L)	276 (217–386.5)	257 (212.5–317.9)	256 (233.7–317.9)	258.3 (195–303.7)
Total protein (g/L)	61.2 (55.6–70.8)	60.1 (57.5–64.2)	59.4 (57.5–62.2)	63.2 (57.6–69)
ALB (g/L)	39.1 (33.5–40.8)	35.4 (32.6–37.5)	35.2 (32.3–37.8)	35.5 (32.2–37.5)
ISA treatment				
Invasive fungal infection^f^, n (%)				
Probable	7 (53.8)	3 (8.6)	3 (15.8)	0 (0)
Proven	0 (0)	27 (77.1)	15 (78.9)	12 (75)
Prophylaxis	6 (46.2)	5 (14.3)	1 (5.3)	4 (25)
Route of administration, n (%)				
Oral only	10 (76.9)	21 (60)	11 (57.9)	10 (62.5)
Intravenous only	3 (23.1)	7 (20)	5 (26.3)	2 (12.5)
Oral and intravenous	0 (0)	7 (20)	3 (15.8)	4 (25)
Initial loading dose^a^	100 (50–200)	200 (200–600)	200 (200–600)	200 (200–200)
Initial maintenance dose^a^	50 (34.8–100)	200 (150–200)	200 (200–200)	200 (100–200)
Duration of treatment (days)^a^	34 (22.5–61)^g^	30 (18.3–56.8)	27.5 (14–49.3)	40.5 (19.8–59)

ALT: alanine aminotransferase; AST: aspartate aminotransferase; ALB, albumin; Cr: creatinine; GGT: gamma glutamyltransferase; LDH: lactate dehydrogenase; RRT: renal replacement therapy; TBil: total bilirubin.

^a^ Data are presented as numbers (%) or medians (IQR).

^b^ including coronary heart disease, pericardial effusion, atrial fibrillation, heart failure and cardiac insufficiency.

^c^ 3 had solid organ transplant and 4 had chronic obstructive pulmonary disease (COPD).

^d^ 2 had kidney transplantation, 3 had continuous venovenous hemofiltration (CVVH) and 1 had continuous venovenous hemodialysis/filtration (CVVHD/F).

^e^ 4 patients were hospitalized in non-ICU, wards at the time of ISA, initiation and were subsequently admitted to the ICU.

^f^ Invasive fungal disease (IFD) was defined as possible, probable, or proven according to EORTC/MSGERC (Donnelly et al., 2020).

^g^ 38% of the patients had missing values due to discharge or transfer to another hospital.

### 3.2 TDM of ISA

**FIGURE 1:**
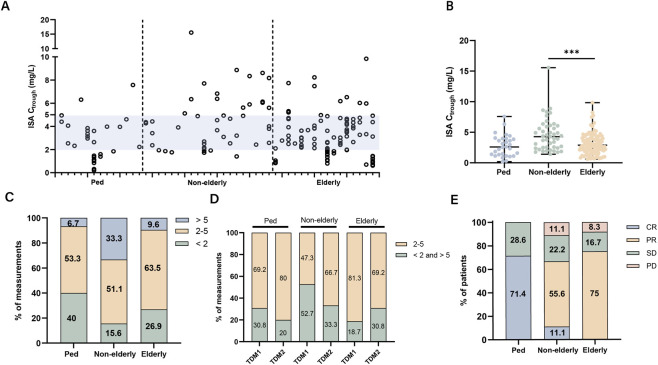
TDM and clinical outcomes of ISA in 48 patients. **(A)** The dispersion of C_trough_ measurements (n = 179) across all patients. **(B)** Comparison of median C_trough_ in each group. **(C)** Distribution range of C_trough_ within each group, categorized as subtherapeutic (<2 mg/L), therapeutic (2–5 mg/L), or supratherapeutic (>5 mg/L). **(D)** The proportions of C_trough_ within the therapeutic range for each group at the time of initial measurement and after dose adjustment. **(E)** Clinical response for each group. CR = complete response, PR = partial response, SD = stable disease, PD = progressive disease.

A total of 179 ISA C_trough_ measurements were performed, with a median (range) of 1 (1–11), 2 (1–7), 5.5 (1–18) measurements per pediatric, non-elderly and elderly patients, respectively (Table 2). The dispersion of TDM levels and number of samples is shown in Figure 1A. Median time of first measurement after initiation in pediatric patients and adult patients were 3.5 days (range 2–10) and 6 days (range 3–15), respectively (Table 2). The median C_trough_ across all age groups were within the therapeutic range (2–5 mg/L). While no statistically significant difference was found between adult patients and pediatric patients (median 2.6 mg/L vs. 3.1 mg/L, *t*-test *P* = 0.065), low-weight pediatric patients (<35 kg) exhibited significantly lower C_trough_ levels than heavier pediatric patients (median 2.3 mg/L vs. 4 mg/L, *t*-test *P* = 0.022). The median C_trough_ in elderly patients are significantly lower than those in non-elderly patients (2.9 mg/L vs. 4.3 mg/L, *t*-test *P* < 0.001) (Figure 1B).

**TABLE 2:** Analysis of 179 plasma C_trough_ of ISA from 48 patients.

TDM parameter	Pediatric patients (n = 30)	Adults		
		Total (n = 149)	Non-elderly patients (n = 45)	Elderly patients (n = 104)
Number of ISA C_trough_ measurement, per patient, median (range)	1 (1–11)	3 (1–18)	2 (1–7)	5.5 (1–18)
First measurement after initiation, days, median (range)	3.5 (2–10)	6 (3–15)	6 (3–14)	7 (3–15)
Initial ISA C_trough,_(mg/L), median (IQR)	4 (2.3–4.2)	3.7 (2.5–4.9)	3.9 (2.3–5.4)	3.2 (2.8–4.1)
Median ISA C_trough,_(mg/L), median (IQR)	2.6 (1.4–3.9)	3.1 (2.1–4.6)	4.3 (2.5–5.9)	2.9 (1.9–4.1)
ISA C_trough_ < 1 mg/L, n (%)	3 (10)	10 (6.7)	0 (0)	10 (9.6)
ISA C_trough_ < 2 mg/L, n (%)	12 (40)	35 (23.5)	7 (15.6)	28 (26.9)
ISA C_trough_: 2–5 mg/L, n (%)	16 (53.3)	89 (59.7)	23 (51.1)	66 (63.5)
ISA C_trough_ > 5 mg/L, n (%)	2 (6.7)	25 (16.8)	15 (33.3)	10 (9.6)
Dosage adjustments, n	4	17	2	15

Overall, in pediatric patients, 14/30 (46.7%) C_trough_ were outside therapeutic range (40% subtherapeutic, 6.7% supratherapeutic). In adults, 60/149 (40.3%) were outside range (23.5% subtherapeutic, 16.8% supratherapeutic). Pediatric patients (40%) and elderly patients (26.9%) tend to have more subtherapeutic levels, while non-elderly patients tend to have more supratherapeutic levels (33.3%) (Table 2). The distribution range of serum concentrations is shown in Figure 1C.

TDM led to 21 total dosage adjustments during the study period (Table 2), the majority of adjustments were dose decreases (14/21, 66.7%). Figure 1D and Table 3 show the distribution of ISA C_trough_ at the time of initial measurement and second monitoring based on TDM. At the time of initial monitoring, the proportions of ISA C_trough_ outside the therapeutic range were 30.8% in pediatric patients, 52.7% in non-elderly patients, and 18.7% in elderly patients. In the second monitoring, the percentage of C_min_ values outside the therapeutic range decreased to 20% in pediatric patients and 33.3% in non-elderly patients.

**TABLE 3:** Analysis of C_trough_ at the time of the first measurement and after the first dose adjustment based on TDM.

TDM parameter	Pediatric patients	Adults		
		Total	Non-elderly	Elderly patients
Initial TDM (n)	13	35	19	16
ISA C_trough,_(mg/L), median (IQR)	4 (2.3–4.6)	3.7 (2.6–4.9)	3.9 (2.3–5.4)	3.2 (2.8–4.1)
ISA C_trough_ < 1 mg/L, n (%)	0 (0)	1 (2.8)	0 (0)	1 (6.2)
ISA C_trough_ < 2 mg/L, n (%)	2 (15.4)	4 (11.4)	4 (21.1)	1 (6.2)
ISA C_trough_: 2–5 mg/L, n (%)	9 (69.2)	22 (62.8)	9 (47.3)	13 (81.3)
ISA C_trough_ > 5 mg/L, n (%)	2 (15.4)	7 (20)	6 (31.6)	1 (6.2)
Second TDM (n)	5	25	12	13
ISA C_trough,_(mg/L), median (IQR)	2.6 (2.5–3.1)	4.4 (2.8–5.3)	4.8 (3.9–6.2)	3.8 (2.2–4.5)
ISA C_trough_ < 1 mg/L, n (%)	0 (0)	0 (0)	0 (0)	0 (0)
ISA C_trough_ < 2 mg/L, n (%)	1 (20)	1 (4)	0 (0)	1 (7.7)
ISA C_trough_: 2–5 mg/L, n (%)	4 (80)	17 (68)	8 (66.7)	9 (69.2)
ISA C_trough_ > 5 mg/L, n (%)	0 (0)	7 (28)	4 (33.3)	3 (23.1)

The coefficient of variation (CV%) of C_trough_ was 51.6% in pediatric patients, 46.8% in non-elderly patients, 33.6% in elderly patients, indicating markedly higher variability in pediatric patients.

### 3.3 Clinical outcomes

Overall, 7 patients (18.9%) had a complete response, 19 patients (51.4%) had a partial response, 8 (21.6%) had stable invasive fungal disease (Table 4). Clinical responses across pediatric, non-elderly, and elderly subgroups are illustrated in Figure 1E Invasive fungal disease progression occurred in three ICU adult patients. Among these cases, one non-elderly patient had an unclear infectious pathogen leading to suboptimal antifungal treatment response, while two patients received RRT. One RRT recipient was an elderly patient who underwent 13 ISA C_trough_ measurements ranging from 0.7 to 4.9 mg/L with a median of 1.2 mg/L. Additionally, we found that in elderly patients, the median C_trough_ in those without a clinical response was below 2 mg/L and was significantly lower than that in those with a clinical response (1.4 mg/L vs. 3.6 mg/L, *t-*test P < 0.001) (Table 4).

**TABLE 4:** Efficacy and safety of ISA in 48 patients.

Parameter	Pediatric patients (n = 7)	Adults		
		Total (n = 30)	Non-elderly patients (n = 18)	Elderly patients (n = 12)
Treatment response, n (%)				
CR	5 (71.4)	2 (6.7)	2 (11.1)	0 (0)
PR	0 (0)	19 (63.3)	10 (55.6)	9 (75)
SD	2 (28.6)	6 (20)	4 (22.2)	2 (16.7)
PD	0 (0)	3 (10)	2 (11.1)	1 (8.3)
Adverse events				
Total, n (%)	1 (7.7)	4 (11.4)	1 (5.3)	3 (18.8)
Diarrhoea, n (%)	0 (0)	1 (2.9)	0 (0)	1 (6.3)
Elevated liver enzymes, n (%)	1 (7.7)	3 (8.6)	1 (5.3)	2 (12.5)
Median ISA C_trough,_(mg/L), CR + PR, median (IQR)	3.3 (2.3–4)	4 (2.8–5)	4.6 (3.6–6.2)	3.6 (2.6–4.5)
Median ISA C_trough,_(mg/L), SD + PD, median (IQR)	4.9 (3.6–4.3)	1.9 (1.3–3.9)	3.9 (2.5–4.9)	1.4 (1–2.5)
Median ISA C_trough,_(mg/L), without adverse events, median (IQR)	2.2 (1.1–4)	3.1 (1.9–4.7)	4 (2.5–5.6)	2.7 (1.6–4.3)
Median ISA C_trough,_(mg/L), with adverse events, median (IQR)	3.4 (3.1–3.6)	3.6 (2.5–4)	7.1 (6.5–7.7)	3.3 (2.5–3.9)

CR, complete response; PR, partial response; SD, stable disease; PD, progressive disease.

5 of the 48 (10.4%) patients experienced adverse events related to ISA (Table 4), with the highest number rate reported in elderly patients (18.8%). One elderly patient experienced diarrhoea (grade 1 by CTCAE) led to discontinuation of the drug, and his median C_trough_ was 2.6 mg/L (range 2.2–3.6). One pediatric patient and three adult patients had elevated liver enzymes (all grade 1 by CTCAE), with their median C_trough_ being 3.4 mg/L (range 3–3.9), 7.1 mg/L (range 5.9–8.3), 3.8 mg/L (range 2.1–8.2), and 4 mg/L (range 2.6–4.6), respectively. We also found that there is no statistically significant difference in the median C_trough_ between patients with and without adverse reactions (*t-*test *P* > 0.5). Overall, our study shows that ISA was used in a diverse patient population and was well tolerated.

### 3.4 Univariate analysis

**FIGURE 2:**
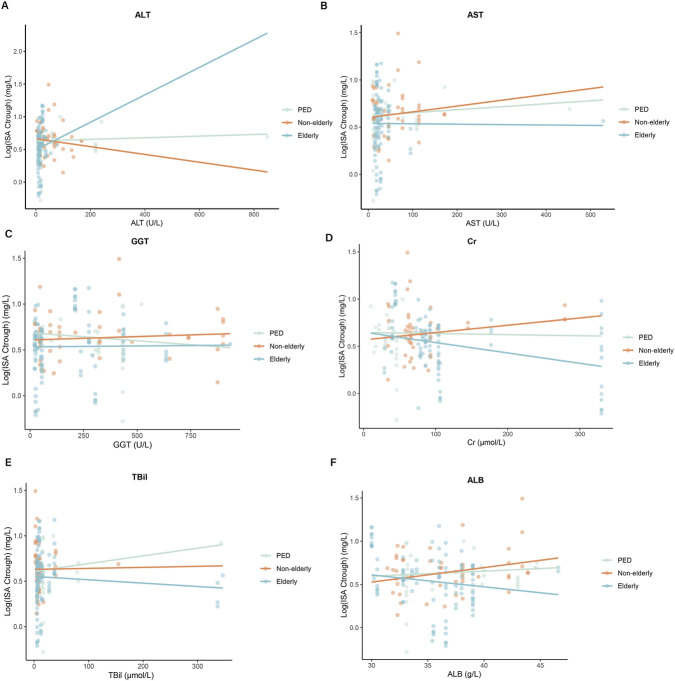
Associations between ISA C_trough_ levels and biochemical covariates. The relationships between **(A)** ALT, **(B)** AST, **(C)** GGT, **(D)** Cr, **(E)** TBil, **(F)** ALB and ISA trough levels. ALT: alanine aminotransferase; AST: aspartate aminotransferase; ALB, albumin; Cr: creatinine; GGT: gamma glutamyltransferase; TBil: total bilirubin.

We first examined the association between ISA C_trough_ levels and clinical covariates (Table 5). Clinical covariates were based on previously reported or hypothesized determinants of ISA exposure, including sex, BMI, ICU admission, and RRT (Tan et al., 2025). To mitigate the bias arising from varying dosages sizes per patient, we normalized the C_trough_ by dose. As shown in Table 5, among pediatric patients, C_trough_ was not associated with gender. Dose-normalized median ISA trough levels were comparable between elderly and non-elderly patients (3.8 (IQR 2.5–5) vs. 4.4 mg/L (IQR 3.1–5.9), *P* = 0.268). In elderly patients, BMI (≤25 or >25 kg/m^2^) showed no significant effect on drug concentrations, while female sex, ICU admission, and RRT were all associated with significantly lower levels (*P* < 0.001). Subsequently, the relationships between biochemical variables and ISA C_trough_ levels were studied. No significant correlation was observed between baseline (ALT, AST, GGT, TBil, Cr, ALB) and ISA C_trough_ levels (P > 0.05 for all) (Figure 2).

**TABLE 5:** Univariable analysis of ISA C_trough_ by Sex, BMI, ICU Admission, and RRT.

Variable	ISA C_trough_						Dose-normalised ISA C_trough_					
​	Pediatric patients (n = 13)		Non-elderly patients (n = 19)		Elderly patients (n = 16)		Pediatric patients (n = 13)		Non-elderly patients (n = 19)		Elderly patients (n = 16)	
​	median (IQR)	*P-*value	median (IQR)	*P-*value	median (IQR)	*P-*value	median (IQR)	*P-*value	median (IQR)	*P-*value	median (IQR)	*P-*value
Sex												
Male	1.7 (1.1–3.9)	0.129	4.6 (3.2–6.1)	**0.008**	3.4 (2.4–4.4)	**<0.001**	4 (2.8–5.6)	0.579	4.6 (3.4–6.1)	0.409	4.2 (3.1–6)	**<0.001**
Female	3.5 (3.1–3.9)		2.2 (1.9–2.7)		2 (1.5–2.7)		3.7 (3.3–4.1)		3.9 (3.5–5)		2.7 (1.8–4.2)	
BMI												
BMI≤ 25 kg/m^2^	NA	NA	3.7 (2.3–5)	0.071	2.8 (1.9–3.9)	0.097	NA	NA	4.3 (3.2–5.2)	0.220	3.8 (2.4–5.2)	0.625
BMI> 25 kg/m^2^	NA		5.6 (3.8–6.4)		3.4 (2.7–5.2)		NA		5.6 (3.8–6.4)		3.4 (2.7–4.4)	
ICU admission												
no	NA	NA	3.9 (2.4–4.4)	0.206	3.6 (2.8–4.5)	**<0.001**	NA	NA	3.9 (2.6–4.4)	**0.049**	4.4 (3.6–7.4)	**<0.001**
yes	NA		4.8 (2.5–6.4)		2 (1.2–3.1)		NA		5 (3.6–6.4)		2.8 (1.7–4.4)	
RRT												
no	NA	NA	4 (2.5–5.6)	0.181	3.6 (2.5–4.5)	**<0.001**	NA	NA	4.3 (4.9–6.1)	0.280	4.1 (3.2–5.9)	**<0.001**
yes	NA		6.1 (4.9–6.1)		1.7 (1.2–2.5)		NA		6.1 (4.9–6.1)		2.6 (1.7–4.3)	

Bold values indicate statistical significance (P < 0.05).

## 4 Discussion

Although ISA is approved in China for the treatment of invasive aspergillosis and mucormycosis in adults, its favorable antifungal efficacy and safety profile have prompted its expanded use in vulnerable populations, including pediatric patients and the elderly patients. This study characterizes real-world TDM of ISA across these age groups in Chinese patients. Analysis revealed pediatric patients exhibited the highest inter-individual variability and a pronounced tendency for subtherapeutic concentrations. TDM-guided dose adjustments effectively reduced the proportion of patients with concentrations outside the therapeutic range, particularly in pediatric and non-elderly patients. Female sex, ICU admission, and RRT were identified as factors associated with significantly lower ISA levels in elderly patients, whereas liver enzyme levels did not correlate with drug exposure.

Our results show intra-individual variability was higher in pediatric patients (51.6%) compared to adult patients (46.8% in non-elderly and 33.6% in elderly patients adults, respectively), which is consistent with previous reports (Borman et al., 2020; Gomez-Lopez et al., 2023). In addition, 46.7% (14/30) of pediatric C_trough_ fell outside the therapeutic range, predominantly subtherapeutic (40%). We found that this trend was more pronounced in younger and lower-weight pediatric patients, with 44% of those <35 kg showing subtherapeutic levels *versus* only 20% in heavier counterparts. This suggests that younger, low-weight patients may clear the drug more rapidly, which is in agreement with the findings of Mendoza-Palomar et al. Mendoz et al. (2025) In addition, Decembrino et al. (202) reported subtherapeutic concentrations (median C_trough_: 1.1 mg/L) in pediatric patients <30 kg receiving 100 mg, while Kennedy et al., 2025 and Segers et al. (2024) demonstrated younger pediatric patients require approximately 1.5–2 times the mg/kg dose to achieve target exposure. Given the established correlation between low exposure and increased mortality risk Elhence et al. (2022), TDM is warranted to guide dose optimization in pediatric patients.

Due to the prolonged half-life of ISA, if TDM is employed, concentrations can theoretically be collected at any point in the dosing interval once a patient has achieved a steady state (approximately 5–7 days with a loading dose and 10–14 days without a loading dose) (McCreary et al., 2023). However, in our study, the time to first TDM after treatment initiation was 3.5 days (2–10 days) in pediatric patients, 6 (3–14) in non-elderly patients, and 7 (3–15) in elderly patients. This is because, in clinical practice, TDM is often initiated earlier to guide therapy, particularly in pediatric patients, critically ill patients, or those at risk of subtherapeutic exposure. Additionally, Darnaud et al. and Decembrino et al. have suggested that at least one blood sample should be collected before the first maintenance dose of ISA in pediatric patients to enable early estimation of the patient’s most likely pharmacokinetic profile (Decembrino et al., 2020; Darnaud et al., 2018). Notably, variability in sampling timing relative to steady-state attainment may limit the comparability of C_trough_ values within and across age groups (Kosmidis et al., 2020; Furfaro et al., 2019; Gatti et al., 2025).

While Desai et al.(2019) observed 38%–47% higher AUC in older female patients compared to male and younger female counterparts, and Kosmidis et al. (2020) identified advancing age as an independent predictor of supratherapeutic ISA levels (>6.0 mg/L; *P* = 0.012), we observed significantly lower median C_trough_ in elderly patients. This discrepancy may be attributed to inadequate dosing, as 33 out of 104 measurements showed maintenance doses were lower than 200 mg recommended dosage (50, 100 or 150 mg, etc.). After dose normalization to 200mg, there is no significant difference in trough levels between non-elderly patients and elderly patients: median 4.4 mg/L (IQR 3.1–5.9) vs. median 3.8 mg/L (IQR 2.5–5), respectively).

Mean ISA trough concentrations in ICU patients have been shown to be lower than those in the general population (Perez et al., 2023; Kriegl et al., 2022; Abdul-Aziz et al., 2020; Mikulska et al., 2024). However, this finding was observed only in the elderly population. It also should be noted that trough concentrations in ICU elderly patients were within the therapeutic range. A possible explanation is that as age increases, the plasma protein binding rate of the drug decreases (Fu et al., 2024), and the resulting alteration in protein binding leads to enhanced drug clearance (Bertram et al., 2023; Ulldemolins et al., 2011). RRT may further complicate ISA pharmacokinetics in critically ill patients. RRT can further reduce ISA blood levels, particularly in critically ill patients with hypoalbuminemia, by enhancing the removal of this highly protein-bound antimicrobial (Pea et al., 2007; Wu et al., 2018). Adsorption to the dialysis membrane represents another potential mechanism (Macleod et al., 2005; Honore et al., 2014). However, the relevance of transmembrane clearance appears limited for ISA, as it accounts for only 0.7% of total clearance (Biagi et al., 2019). Critically ill elderly patients who receiving RRT in our cohort tended to have lower ISA blood levels.

Variations in geographic regions and patient characteristics contribute to differences in drug metabolism. Zhang et al. (2021) reported that the clearance of ISA in Chinese healthy volunteers was approximately 40% lower than in Western healthy volunteers (1.66 L/h *versus* 2.57 L/h), resulting in an almost 50% higher exposure. Similarly, a population pharmacokinetic (PPK) model developed from pooled data of nine Phase 1 studies and the Phase 3 SECURE trial showed that Asians have approximately 36% lower clearance compared to Caucasians (De et al., 2016).The fact that a significant portion of our patients, particularly non-elderly patients, achieved ISA C_trough_ levels at supratherapeutic levels (with 33.3% > 5 mg/L) aligns with the reported lower clearance in Asian populations.

Regarding safety, ISA demonstrated a favorable tolerability profile in both pediatric and elderly populations, with a low incidence of adverse events. The majority of adverse events were mild, further corroborating the favorable safety profile of ISA observed in prior studies (Segers et al., 2024; Arrieta et al., 2021; Ishida et al., 2025). We also did not find any association between ISA concentration and toxicity, as the median C_trough_ were not significantly different between patients with and without adverse reactions. Furthermore, one elderly patient developed voriconazole-related hepatotoxicity, and after switching to ISA, hepatic function recovered.

The sample size in each age group in this study was limited, and the number of patients included in each cohort in the univariate analysis was very small, particularly for the pediatric and RRT cohorts, which may jeopardize the generalizability of our findings to larger patient populations. In addition, this study has several other limitations. Firstly, we could not independently validate the suitability of the 2–5 mg/L therapeutic range for these specific populations. The high proportion of patients outside this range, coupled with adequate clinical response in some with low levels and absence of toxicity in some with high levels, suggests that the optimal target may vary across different patient subgroups. Secondly, our pharmacokinetic evaluation relied solely on total ISA C_trough_ (both bound and unbound ISA), which may not fully reflect the pharmacologically active unbound fraction, especially given ISA’s high plasma protein binding (>99%). Thirdly, the interplay of multiple patient factors (e.g., cardiovascular disorders, organ failure, renal insufficiency, hepatic impairment and CYP3A5 polymorphism (Soman et al., 2025; Miceli et al., 2026)) makes it difficult to attribute differences in C_trough_ solely to the variables we analyzed. Lastly, multivariate analysis could not be performed due to the limited sample size in each age group. This prevented us from identifying potential independent predictors of ISA exposure. Therefore, our findings should be interpreted with caution, and further validation through larger, prospective, multicenter studies is warranted.

## 5 Conclusion

To the best of our knowledge, this is the first study to describe real-world data about the TDM of ISA across different age groups in the Chinese population. ISA exhibits significant variability in exposure among patients of different ages, particularly in pediatric patients. Female sex, ICU admission, and RRT were identified as factors associated with significantly lower ISA levels in elderly patients. Future prospective studies with larger sample sizes are warranted to establish reliable exposure–response relationships and refine dosing recommendations in Chinese pediatric and elderly patients.

## Data Availability

The original contributions presented in the study are included in the article/supplementary material, further inquiries can be directed to the corresponding author.
